# Minimally Invasive Resection of Benign Osseous Tumors of the Spinal Column: 10 Years’ Experience and Long-Term Outcomes of a Specialized Center

**DOI:** 10.3390/medicina58121840

**Published:** 2022-12-15

**Authors:** Khalil Salame, Zvi Lidar, Morsi Khashan, Dror Ofir, Gilad J. Regev

**Affiliations:** Spine Surgery Unit, Department of Neurosurgery, Tel Aviv Sourasky Medical Center, and Sackler Faculty of Medicine, Tel Aviv University, Weitzman 6, Tel Aviv 69978, Israel

**Keywords:** benign vertebral tumors, osteoblastoma, osteoid osteoma, primary spine tumors, surgical technique

## Abstract

*Background and Objectives*: Benign osseous tumors of the spinal column comprise about 10% of all spinal tumors and are rare cause for surgery. However, these tumors pose various management challenges and conventional surgery may be associated with significant morbidity. Previous reports on minimally invasive resection of these lesions are rare. We report a series of patients managed by total resection of benign osseous spine tumors using MIS techniques. Surgical decisions and technical considerations are discussed. *Materials and Methods*: A retrospective evaluation of prospectively collected data of patients who underwent minimally invasive surgery for removal of benign osseous vertebral tumors. Demographic, clinical and radiographic features, operative details and final pathological reports were summarized. Primary outcomes were completeness of tumor resection and pain relief assessed by VAS for back and leg pain. Secondary outcome measures were recurrence of tumor on repeat post-operative MRI and postoperative unstable deformity on standing scoliosis X-rays. *Results*: This series included 32 cases of primary osseous spine tumors resected by minimally invasive techniques. There were 17 males and 15 females aged 5–68 years (mean 23.3). The follow-up period was 8–90 months (mean 32 months) and the preoperative symptoms duration was 9–96 months. Axial spinal pain was the presenting symptom in all the patients. Five patients also complained about radicular pain and four patients had antalgic scoliosis. The tumor involved the thoracic spine in 12 cases, the lumbar segment in 11, the cervical in 5 and the sacral area in 4 cases. Complete tumor removal was performed in all patients. No procedure-related complications were encountered. Histopathology showed osteoid osteoma in 24 patients, osteoblastoma in 5 patients, and fibrous dysplasia, fibroadenoma and eosinophilic granuloma in one case each. All patients experienced significant pain relief after surgery, and had stopped pain medications by 12 months postoperatively. No patient suffered from tumor recurrence or spinal deformity. *Conclusions*: Minimally invasive surgery is feasible for total removal of selected benign vertebral tumors and may have some advantages over conventional surgical techniques.

## 1. Introduction

The vast majority of vertebral tumors are metastatic, usually affecting the older population. Primary benign tumors of the spinal column are rare, accounting for 10% of all spinal neoplasm, are more common in children and young adults, and are uncommon cause for surgery. The highest incidence is in the lumbar region, followed by the thoracic and cervical segments. The lesion may be located in any part of the vertebra, although the posterior vertebral arch is more involved.

Pain is the most common presenting symptom, followed by spinal deformity, and up to 23% of the pediatric patients with these tumors have scoliosis at the time of presentation [1,2,3,4]. Management approaches vary from percutaneous ablative modalities to gross complete removal with or without spinal fixation [5,6]. However, the conventional surgical resection of these lesions often entails intralesional curettage of the lesion until normal vertebral bone is reached. Complete removal is often challenging, since intraoperative localization of the these lesions is usually tricky, especially in the thoracic spine. The tumor is usually hidden within normal bone and therefore is not identifiable on the bone surface. In addition, the size of these tumors is often very small, making exposure more difficult. CT navigation is effective for accurate localization of the lesion, choosing the best trajectory and evaluation of the borders of resection [7,8], but this modality should be used cautiously in young children. For all these features, extensive techniques were traditionally required for the removal of benign vertebral tumors. Although conventional open surgery allows resection of the tumor, it usually requires extensive soft tissue dissection and removal of normal vertebral parts, and may be associated with high morbidity. The conventional surgical approach can lead to intra-operative blood loss, severe pain, a prolonged recovery period and spinal instability that necessitates instrumented fusion.

Compared to conventional surgery, minimally invasive techniques have the benefits of smaller skin incision, less bleeding, less soft tissue damage, lower rate of infection, reduced postoperative pain and shorter recovery period. Minimally invasive spine surgery has some drawbacks such as higher utilization of fluoroscopy, slightly higher incidence of dural tears and longer operative time, but these are significant only in the learning phase [9,10,11,12,13]. This is a case series of patients with benign vertebral tumors who underwent surgical removal using the minimally invasive technique system through a variety of surgical approaches, according to the exact tumor location.

Previous studies of benign osseous spine tumors managed by minimally invasive surgery are scarce [14,15,16]. Some authors have focused on intradural extramedullary tumors, metastatic extradural tumors, and tumors with mixed intradural and extradural involvement, and have rarely addressed intramedullary tumors [17,18,19,20,21]. The current study describes our experience in minimally invasive techniques for complete removal of benign primary vertebral tumors with long term follow up. Our approach is different from the percutaneous minimally invasive ablation procedures. This is the largest series in the literature on minimally invasive surgery for benign vertebral tumors.

## 2. Methods

This is a retrospective review of prospectively collected data. We included all patients with benign primary vertebral tumors who underwent surgical resection using minimally invasive techniques. The study was approved by the hospital ethical committee.

The patients’ electronic files were reviewed for demographic parameters, clinical manifestations and radiographic features, focusing on the spinal level and exact location in the vertebra. The operative time, blood loss, hospital length of stay, pathological report and postoperative complications were also collected.

Follow up of all patients was recorded at 1, 3, and 6 months after surgery, and then at 12-month intervals. Patients underwent complete neurological examination and imaging studies were evaluated for tumor recurrence and spinal deformity. Complete neurological examination was performed in all patients preoperatively, at discharge and in follow-up visits.

The primary outcomes were completeness of tumor resection and resolution of axial back pain as evaluated by the Visual Analog Scale VAS and the Numerical Rating (NRS) in patients and children older than 7 (Wong Baker Faces Pain Rating Scale was used in children of 3–7 years’ age). Secondary outcomes were tumor recurrence, evaluated by follow-up MRI scans, and spinal instability, evaluated by plain X-rays in the standing position.

### Surgical Technique

The minimally invasive approach was utilized for tumors with benign clinical and imaging features. The maximal tumor diameter was up to 30 mm; larger tumors were operated on using a conventional open technique. This is based on our previous experience, but we found no reference to this in the literature. Open surgery was also elected for lesions with high vascularity, since it allows better control of intraoperative bleeding. For lesions suspected as malignant, a percutaneous biopsy under CT guidance and systemic evaluation were completed before decision on the most appropriate management.

**Figure 1:**
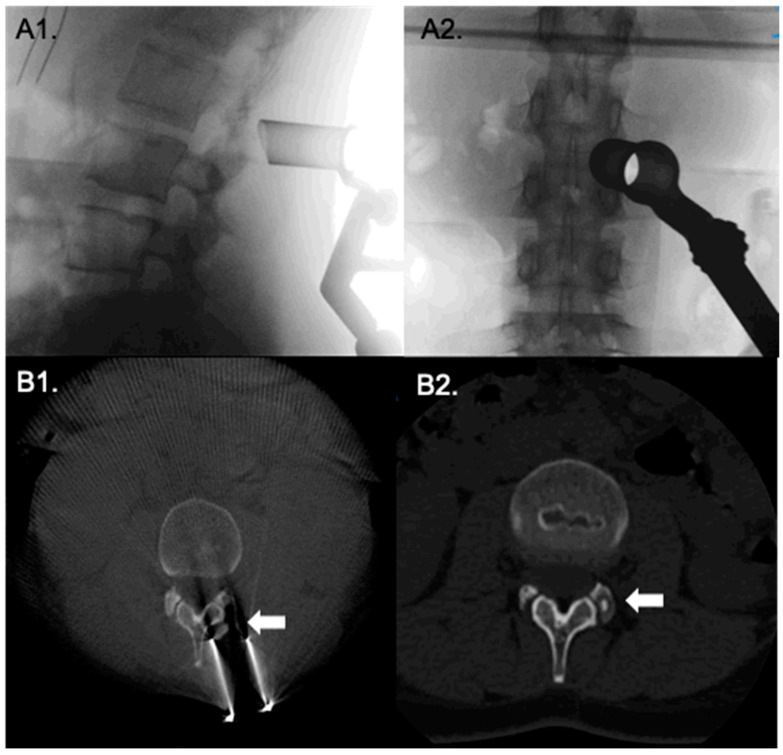
Direct posterior approach to to osteoid osteoma in the facet joint. Intraoperative fluoroscopy, (**A1**,**A2**), verify correct positioning of the tubular retractor. (**B1**,**B2**) show intraoperative O-Arm CT views before and after tumor removal.

Surgery was performed under general endotracheal anesthesia. The patient was positioned prone and fluoroscopy was used to choose the entry site and trajectory of the approach. The skin incision was 18–20 mm long, about 10 mm off midline on the same side, as the lesion is deepened across the fascia. We used the METRx system (Medtronic Sofamor Danek, Memphis, TN, USA). Dilators of increasing diameter up to 20 mm were sequentially inserted through the paravertebral muscles. Then, a tubular retractor with a suitable diameter and length allowing access to the lesion was inserted and secured to the table. The position of the retractor was verified with fluoroscopy in lateral and antero-posterior views (Figure 1). The next steps were performed under the surgical microscope. Electrocautery was used to expose the bony elements.

Bone removal was performed using a high-speed drill and completed with kerrison rongeurs. The tumor was exposed and resected with pituitary forceps and curettes of different sizes. After hemostasis and irrigation, the tubular retractor was removed. The fascia was closed with vicryl sutures. This is better performed under a microscope, especially in obese patients. The skin was closed with intradermal sutures or biologic glue.

Modifications of the surgical technique might be required in each case according to anatomical characteristics and relationships of the lesion.

## 3. Results

Thirty-two patients underwent minimally invasive surgery for removal of benign ossoeous spine tumors. The study cohort included 17 males and 15 females aged 5 to 68 (mean 23.3) years. The mean follow-up was 32 months (8–90). All the patients suffered from axial spinal pain; five patients also complained about radicular pain.

Antalgic scoliosis was detected before surgery in four patients and resolved by 12 months postoperatively. All patients were neurologically intact. The duration of symptoms until surgery was 9 months to 8 years. Axial pain was the presenting symptom in all the patients, and they received medical treatment with NSAIDs and salicylic acid. Nine patients reported persistent pain despite treatment, four patients complained about gastrointestinal intolerance, one patient developed disturbances of renal function, one showed multiple drug allergy, and the remaining patients preferred surgical management over permanent medications.

Spinal level of the tumor: the tumor location was cervical in 5 cases, thoracic in 12 cases, in the lumbar vertebra in 11 cases and in the sacral in 4 cases.

As for the exact tumor location within the involved vertebra, the most common location was at the lamina–facet junction in 11 cases. The lesion was confined to the vertebral body in six cases, confined to the lamina in five cases, inside the pedicle in three cases, and confined to the facet joint in two cases. The lesion involved the facet–intervertebral foramen in two cases, the facet–transverse process in one case, the vertebral body–intervertebral foramen in one case, and the transverse process–rib head in one case.

**Figure 2:**
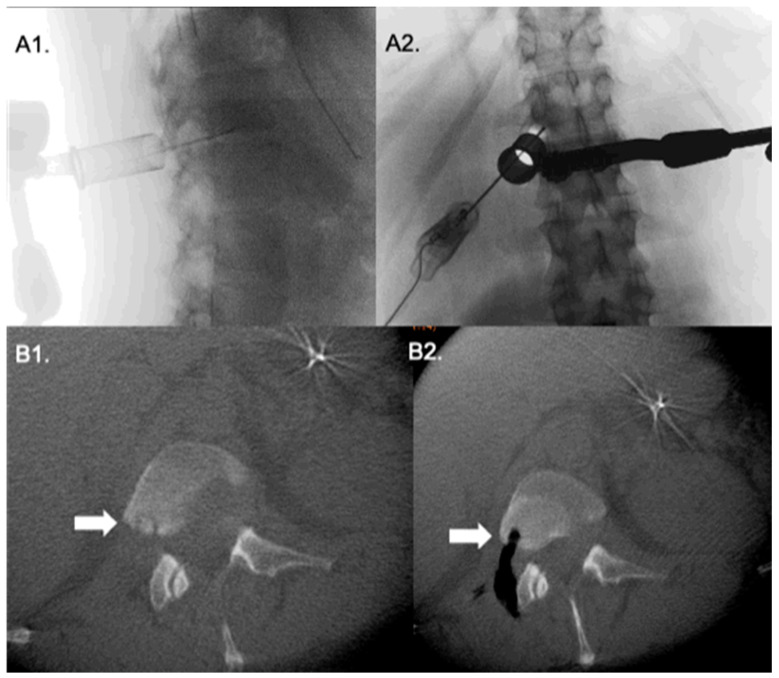
Transforaminal approach to to a tumor in the posterior aspect of L1 vertebral body. Lateral and antero-posterior fluoroscopy (**A1**,**A2**) show the tubular retractor with a probe pointing to the lesion site. Intraoperative O-arm CT axial cuts (**B1**,**B2**) before and after tumor resection.

**Figure 3:**
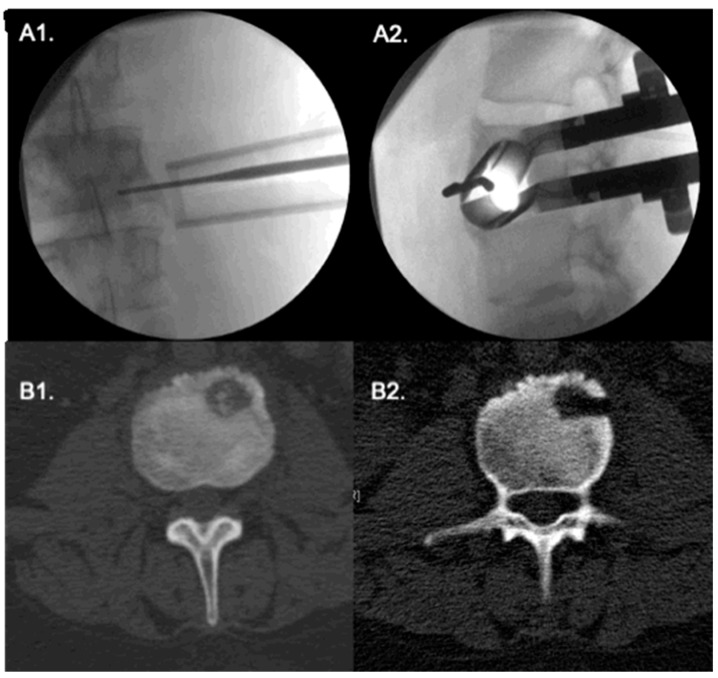
Extreme lateral approach to an L3 vertebral body tumor. (**A1**,**A2**) Antero-posterior and lateral fluroscopy show the correct positioning of tubular retractor; the probe points to the tumor. (**B1**,**B2**) intraoperative CT show the tumor. A pituitary forceps is shown inside the lesion.

Surgical results: The surgical approach was modified according to the anatomical location of the tumor. Tumors in the posterior vertebral elements were removed by a direct posterior approach (Figure 1). The transpedicular path was used for tumors located in the pedicle. For tumors situated in the postero-lateral aspect of the vertebral body, we used a transforaminal approach (Figure 2). In one case, with a tumor sited at the central dorsal part of the S1 vertebral body, we performed hemilaminotomy and the dural sac was retracted to reach the tumor through the spinal canal. On the other hand, a tumor sited in the anterior part of the lumbar vertebral body was removed through a lateral trans-psoas approach to the lumbar spine (Figure 3).

Surgery was performed with O-Arm navigation in 11 cases. One of these patients suffered from a cervical tumor kissing the wall of the transverse process, and the navigation allowed safe removal of the tumor, avoiding injury of the vertebral artery.

Total resection of the tumor was performed in all cases. Blood loss was less than 50 mL and the operative time ranged from 40 to 130 min (mean 70). There was no significant violation of spinal stability in any of the cases, thus fixation was not required. All patients were discharged at 1–3 days after surgery (mean 1.6 days).

The histological diagnosis revealed osteoid osteoma in 24 cases, osteobalstoma in 5, and fibrous dysplasia, fibroadenoma and eosinophilic granuloma in one case each.

Pain outcome: All patients experienced significant pain resolution and the average pain score dropped from 8 (7–9) to 0.8 (0–3) following surgery. All patients were weaned from pain medications within 12 months after surgery. Until the present day, none of the patients suffered tumor recurrence or postoperative deformity. The clinical and radiographic outcomes were not influenced by the tumor histology.

## 4. Discussion

This is a case series of 32 patients with primary benign vertebral tumors treated by complete resection using tubular minimally invasive techniques. Benign vertebral tumors, are rare comprising only 4% to 13% of all spinal tumors.

The preferred management for these tumors is complete surgical resection with intention for cure [1,5]. This is in contrast to the approach for tumors with malignant appearance, in which a percutaneous biopsy under CT or fluoroscopy is undertaken, followed by systemic evaluation, in order to plan the best management, and where multidisciplinary team work is required [22,23].

Surgical removal of vertebral tumors may be technically challenging, especially for small lesions located within the vertebral body. The lesion is usually surrounded by normal bone and not identified on the surface of the vertebral bone; thus, intraoperative localization of the tumor is often tricky. Conventional open surgery often entails significant dissection of normal soft tissue and removal of normal bony structures, which may necessitate instrumented fusion. Extensive lateral or anterior approaches may be needed, especially in the thoracic or retroperitoneal compartments. Extensive procedures are statistically associated with higher risk of neural and vascular complications.

The growing experience in minimally invasive surgery, supported by the technological development of equipment, enabled significant improvement in the care of patients suffering from spinal tumors, whether benign or malignant. Using these techniques, the tumor can be resected with limited violation of the surrounding musculoskeletal structures. As a result, the risk of iatrogenic spinal instability and the need for spinal fixation are avoided. Iatrogenic instability is common when the tumor involves the facet joint, as in the majority of our cases; however, due to limited violation of the facet joints in the MIS, none of the patients developed instability or fracture. Patients experienced less postoperative pain and consumed smaller doses of drugs, particularly narcotics. Other advantages include lower complication rate and shorter hospitalization time and recovery period [9,10,11,12].

Minimally invasive and mini-open techniques have been used in surgery on benign intradural spinal tumors [18,22], while earlier publications on resection of vertebral tumors are limited [14,15,16].

In the current series, the most common tumors were osteoid osteoma and osteoblastoma. About 40% of osteoblastoma and 25% of osteoid osteoma cases appear in the spinal column, and together they comprise almost 3% of all primary bone tumors.

Although classified as benign, osteoblastoma has aggressive behavior with risk of malignant transformation to osteosarcoma [24,25]. Osteoid osteoma is a benign tumor with latent course that may even regress and involute overtime; therefore, follow up with symptomatic management can be reasonable in selected patients. However, there have been reports on osteoid osteoma transforming into osteoblastoma and even into high-grade malignant osteosarcoma [26,27]. Surgical resection is indicated in cases of osteoblastoma or osteoid osteoma manifested by persistent symptoms, and is curative.

However, other modalities are available for the management of benign vertebral tumors, including percutaneous radiofrequency (RF) ablation cryoablation, laser thermal ablation and microwave ablation [28]. Radiofrequency is probably the most commonly used modality, but has some limitations, especially when the tumor is very close to neural or vascular structures. The risk of thermal cytotoxicity to the spinal cord and nerve roots must be weighed carefully before any spinal RF ablation treatment [29,30]. Yu et al. recommended that RF be applied only if the spinal cord and nerve roots are separated from the tumor by CSF, in order to avoid neurological complications [31]. Noel et al. reported high technical and clinical success in CT-guided RF ablation of osteoid osteoma in children by using neurophysiological monitoring with progressive time and temperature protocol [32]. In addition, there is the possibility for relapse of the spinal tumors after RF ablation treatment; thus, surgery or additional ablation procedures might be required. In laser photocoagulation, infrared light is transmitted via optic fibers to induce fast heating, which causes protein denaturation and coagulation necrosis of tumor cells. An advantage of laser ablation is the predictability of the ablation area size in relation to the delivered energy, but the ablation zone is not demonstrated in CT [33]. Contrary to this mechanism, cryoablation is based on rapid cooling, which causes osmosis, leading to tumor cell dehydration and death. Cryoablation is suitable for benign tumors with large soft tissue components and for osteoblastic tumors of the posterior vertebral elements [34]. Microwave ablation, in which heat is delivered to induce tissue coagulation necrosis, was found to be effective for metastatic spine tumors, but has not been used for benign tumors. In addition, the margins of the ablation zone are ill-defined and overheating may damage adjacent neural tissues [35].

In the current series, complete marginal tumor removal was achieved in all patients, who remained disease free in their last follow-up visits.

It should be noted that four of our patients presented with scoliosis that resolved during the postoperative follow up, but no patient developed iatrogenic deformity. This differs from the large study by Saifuddin et al. [4] on 465 cases of osteoid osteoma and osteoblastoma, 44 museum cases and 421 from the literature, which found scoliosis in 63% of cases, with the lesion usually on the concave side of the curve.

The minimally invasive approach described here differs from conventional open surgery in a few technical aspects. Exact placement of the tubular retractor before beginning the tumor removal allows the shortest, most accurate trajectory, and is the key for successful surgery. Therefore, meticulous preoperative planning cannot be overemphasized. The exact tumor location, size and relationships with adjacent neural and vascular structures, as well as the patient’s body habitus, should be carefully evaluated. In the minimally invasive approach, the surgeon’s visual field is rather limited, which may inhibit complete tumor resection. In order to avoid this pitfall, the tumor size relative to tubular retractor diameter and the anatomical spinal components have to be assessed before starting the tumor excision. In addition, the tubular retractor often has to be adjusted through the procedure to enable appropriate access to the entire perimeter of the tumor.

The use of intraoperative navigation provides higher precision, which allows complete tumor excision and minimizes injury to the neighboring tissues [7,8]. Yet, it should be used cautiously to reduce exposure of the patient and staff to radiation, particularly in younger children.

In this series, we used different surgical approaches as dictated by the location of the tumor and the involved vertebral parts: tumors of the posterior vertebral elements were resected by a direct posterior approach; tumors in the pedicle by the trans-pedicular approach with preservation of the pedicle cortex; tumors at the posterolateral part of the vertebral body through a transforaminal approach; tumors in the anterior part of the vertebral body by an extreme lateral trans-psoas retroperitoneal approach; and for tumors in the midline posterior aspect of the vertebral body, tumors inside the spinal canal or for decompression of the thecal sac, the approach was trans-spinal canal.

The minimally invasive technique may not be suitable in some situations. Firstly, for suspected malignant tumors, a CT-guided biopsy should be performed to determine the best treatment and prevent tumor spread, thus keeping the option of radical intra-lesional or en-bloc resection if indicated. Secondly, for large tumors, whether benign or malignant, open or mini-open approaches should be chosen. For tumors with rich vascularity, preoperative angiography and closure of supplying vessels should be considered, and an open approach might be preferred since the ability to control profuse bleeding is hindered with minimally invasive exposure.

## 5. Limitations

The limitations of our study include its retrospective design and lack of a control group of patients operated on by other techniques or managed by different modalities. For some of the patients, the follow-up period is still short. The number of patients is small, although to the best of our knowledge this is the largest series in the English-language literature. A larger series with longer follow up and comparative analysis with other modalities are needed to establish the advantages of this technique.

## 6. Conclusions

To the best of our knowledge, this is the largest series on minimally invasive resection of benign osseous spine tumors. Complete removal was achieved in all cases without surgical complications. Our experience suggests that the minimally invasive technique might be a valuable option for the surgical management of select benign extradural tumors.

## Data Availability

Not applicable.
